# Insights into the structure–photoreactivity relationships in well-defined perovskite ferroelectric KNbO_3_ nanowires†
†Electronic supplementary information (ESI) available: Additional figures and tables, synthetic procedures, theoretical calculation methods, experimental details for XRD, spectroscopy (XPS, XANES and UV-vis), microscopy (AFM, SEM, HRTEM and STEM) and photocatalytic experiments. See DOI: 10.1039/c5sc00766f


**DOI:** 10.1039/c5sc00766f

**Published:** 2015-04-23

**Authors:** Tingting Zhang, Wanying Lei, Ping Liu, José A. Rodriguez, Jiaguo Yu, Yang Qi, Gang Liu, Minghua Liu

**Affiliations:** a National Center for Nanoscience and Technology , Beijing 100190 , China . Email: liug@nanoctr.cn ; Email: liuminghua@nanoctr.cn; b Institute of Materials Physics and Chemistry , School of Sciences , Northeastern University , Shenyang 110004 , China; c Chemistry Department , Brookhaven National Laboratory , Upton , New York 11973 , USA; d State Key Laboratory of Advance Technology for Material Synthesis and Processing , Wuhan University of Technology , Wuhan 430070 , China

## Abstract

Structure–function correlations are a central theme in heterogeneous (photo)catalysis. In this study, the geometric and electronic structure of perovskite ferroelectric KNbO_3_ nanowires with respective orthorhombic and monoclinic polymorphs have been systematically addressed. By virtue of aberration-corrected scanning transmission electron microscopy, we directly visualize surface photocatalytic active sites, measure local atomic displacements at an accuracy of several picometers, and quantify ferroelectric polarization combined with first-principles calculations. The photoreactivity of the as-prepared KNbO_3_ nanowires is assessed toward aqueous rhodamine B degradation under UV light. A synergy between the ferroelectric polarization and electronic structure in photoreactivity enhancement is uncovered, which accounts for the prominent reactivity order: orthorhombic > monoclinic. Additionally, by identifying new photocatalytic products, rhodamine B degradation pathways involving *N*-deethylation and conjugated structure cleavage are proposed. Our findings not only provide new insights into the structure–photoreactivity relationships in perovskite ferroelectric photocatalysts, but also have broad implications in perovskite-based water splitting and photovoltaics, among others.

## Abstract 2

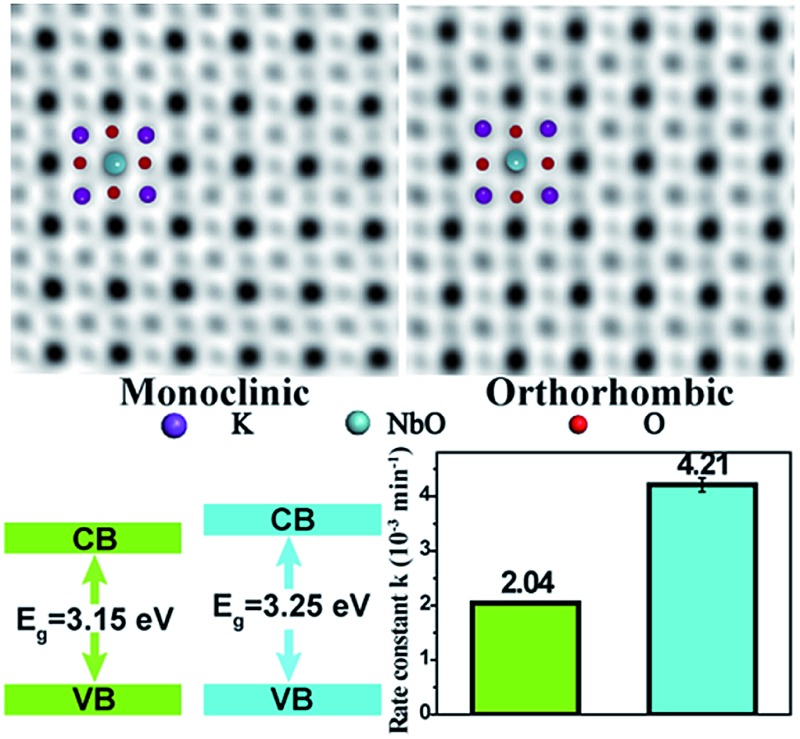
1D perovskite-type orthorhombic KNbO_3_ nanowires display RhB photodegradation about two-fold as large as their monoclinic counterparts and a synergy between ferroelectric polarization and electronic structure in photoreactivity enhancement is uncovered.

## Introduction

Since Fujishima and Honda discovered photocatalytic water splitting on TiO_2_,^1^ the past decades have seen a significant rise in light-driven pollutant abatement,^2^–^5^ selective oxidation,^6^,^7^ and water splitting.^8^–^14^ Despite tremendous progress being made, the mechanisms of photocatalysis are not yet known in detail. In the conceptual framework of heterogeneous (photo)catalysis, the mechanistic understanding of structure–function relationships is a prerequisite to the rational design of efficient photocatalysts. On the other hand, semiconductor-based photocatalysis is inherently complex in that both the surface structure and bulk structure of a given photocatalyst synergistically dictate the photocatalytic efficiency:^5^,^15^ the bulk absorbs incident photons and generates e^–^–h^+^ pairs, while the surface harnesses available e^–^–h^+^ to catalyze target adsorbates at the photocatalytic active sites. To this end, a driving force (*e.g.*, internal electronic field functional like a p–n junction) for facilitating the spatial separation of the e^–^–h^+^ pairs is desirable.^16^–^21^ Apparently, the complexity of photocatalysis hampers an atomistic understanding of structure–function relationships. For instance, most previous studies about internal electronic field-mediated photocatalysis are carried out on irregularly-shaped powders in which the active sites are unclear,^22^–^24^ making it difficult to explore structure–function relationships. Only a thorough understanding of structure–function relationships in well-defined model catalysts can add new dimensions to our fundamental view of “real world” catalysis as well as rationally design catalysts at an atomic-level.^25^–^27^ Recent advances in synthesizing nanocrystals allow for fine control of the size and shape of (photo)catalysts.^28^–^31^ To date, a great deal of studies on nanostructured photocatalysts are focused on facet-dependent photocatalysis.^32^–^38^ Nevertheless, the underlying synergetic effects involved in photocatalysis remain largely unexplored.

In this study, we investigated the photocatalytic degradation of rhodamine B (RhB) in water (a model reaction in the removal of organic pollutants from waste water) by one-dimensional (1D) single-crystalline potassium niobate (KNbO_3_) nanowires (NWs) with respective orthorhombic and monoclinic polymorphs. KNbO_3_ is a typical ferroelectric perovskite (general formula ABO_3_, where A is a metal, B is a second metal, and O is oxygen) with diverse emerging technological applications, including photocatalysis, with the advantages of non-toxicity, cost-effectiveness and high stability under light illumination.^39^,^40^ In 1D nanostructures, it is possible to enhance the photoreactivity by tuning the transport of photogenerated charge carriers through quantum confinement.^41^,^42^ Currently, the understanding of ferroelectric materials is based primarily on theory, since few experimental techniques can be used to probe the local atomic displacements that give rise to polarization. Thanks to recent progress in aberration-corrected transmission electron microscopy (TEM),^43^,^44^ it is possible to measure local polarization displacements at an accuracy of several picometers and determine surface terminations by profile imaging. Herein, using advanced aberration-corrected scanning transmission electron microscopy (STEM), we directly visualize surface photocatalytic active sites, measure local atomic displacements at an accuracy of several picometers, and quantify ferroelectric polarization combined with spin-polarized density functional theory (DFT) calculations. We uncover a novel photocatalytic synergy between ferroelectric polarization and electronic structure, which accounts for the prominent reactivity order: orthorhombic > monoclinic. Additionally, RhB degradation pathways involving *N*-deethylation and conjugated structure cleavage are proposed.

## Results and discussion

**Fig. 1:**
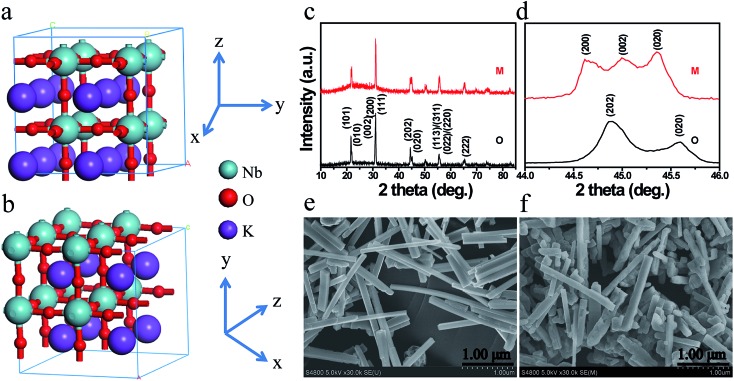
(a) Crystal structure of *m*-KNbO_3_. (b) Crystal structure of *o*-KNbO_3_. (c) Powder XRD patterns of *m*- and *o*-KNbO_3_ NWs. (d) High-resolution XRD patterns in the range of 44°–46°. (e) SEM image of *m*-KNbO_3_ NWs. (f) SEM image of *o*-KNbO_3_ NWs. M: *m*-KNbO_3_ NWs; O: *o*-KNbO_3_ NWs.

Fig. 1a and b illustrate the crystal structures of monoclinic and orthorhombic KNbO_3_ polymorphs, denoted as *m*-KNbO_3_ and *o*-KNbO_3_, respectively. X-ray diffraction (XRD) patterns as displayed in Fig. 1c confirm the as-prepared *m*-KNbO_3_ (space group *P*1*m*1) and *o*-KNbO_3_ (space group *Bmm*2, JCPDS card 71-2171) samples with comparable crystallinity, evidenced by the full-width at half-maximum (FWHM), *e.g.*, about 0.23° for the peak at 31.5° for *m*-KNbO_3_ and *o*-KNbO_3_. High-resolution XRD in the range of 44°–46° (Fig. 1d) further indicates apparent structural differences between the above two structures. Scanning electron microscopy (SEM) images (Fig. 1e and f) show well-defined elongated *m*- and *o*-KNbO_3_ NWs. Combined SEM and atomic force microscopy (AFM) (Fig. S1†) measurements indicate that the average length, width, and height for the *m*-KNbO_3_ NWs are (1.3 ± 0.5) μm, (106 ± 47) nm, and (138 ± 36) nm, respectively, while for the *o*-KNbO_3_ NWs they are (1.2 ± 0.4) μm, (102 ± 27) nm, and (136 ± 17) nm, respectively. High-resolution transmission electron microscopy (HRTEM) images (Fig. S2†) prove that the *m*-KNbO_3_ NWs are enclosed by {100}, {010} and {001} facets,^45^ and the *o*-KNbO_3_ NWs are enclosed by {101} and {010} facets.^46^ The growth direction of the *m*- and *o*-KNbO_3_ NWs is [100] and [101], respectively. The BET specific surface area is measured to be 4.7 and 4.8 m^2^ g^–1^ for the respective *m*- and *o*-KNbO_3_ NWs.

**Fig. 2:**
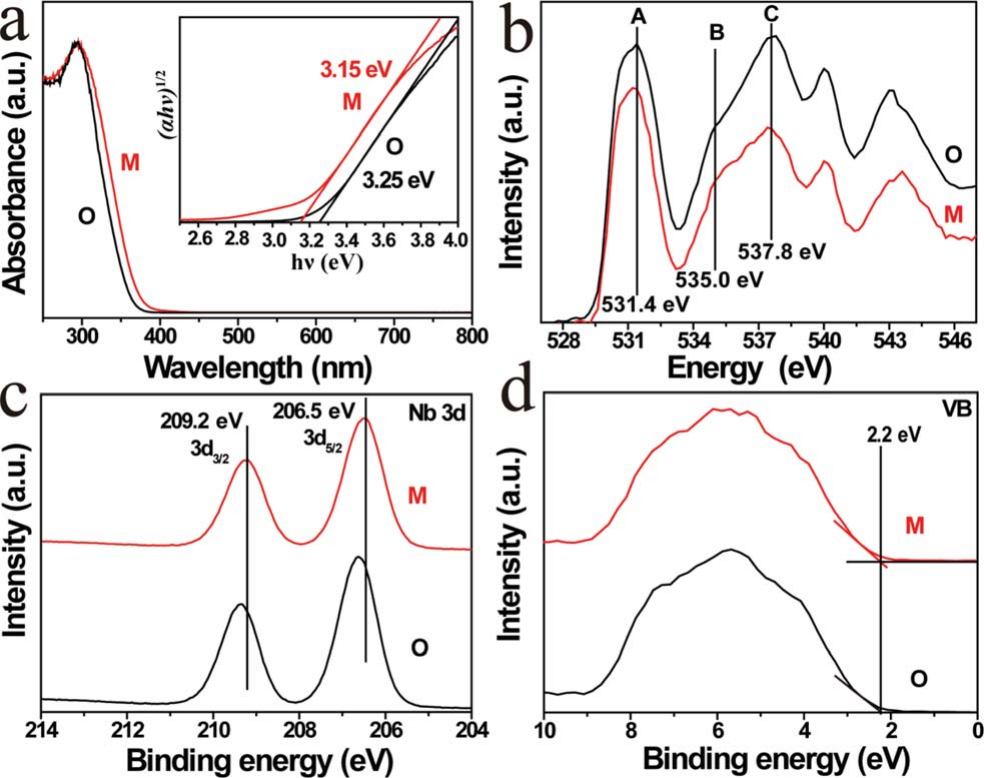
(a) DRUV-vis spectra and corresponding plots of (*αhν*)^1/2^*versus* photon energy (*hν*). (b) O *K*-edge XANES spectra. (c) High-resolution XPS spectra of Nb 3d core-level. (d) Valence band spectra. M: *m*-KNbO_3_ NWs; O: *o*-KNbO_3_ NWs.

The photophysical property is revealed by diffuse-reflectance UV-vis (DRUV-vis) spectroscopy (Fig. 2a). The bandgap (*E*_g_) of the KNbO_3_ NWs is determined using a Tauc plot (in the inset of Fig. 2a): the *m*- and *o*-KNbO_3_ NWs possess a bandgap of 3.15 and 3.25 eV, respectively. The bandgap of *o*-KNbO_3_ is similar to that reported in previous work (3.2 eV).^47^ Peak A centered at 531.4 eV is ascribed to the hybridization of O 2p with Nb 4d–t_2g_ through π* interaction. Both peaks B and C, observed at 535.0 and 537.8 eV respectively, are attributed to the hybridization of O 2p with Nb 4d–e_g_ (σ* interaction) and Nb 5p (π*, σ* interaction).^48^ The relative peak intensity ratio between A and C is dramatically decreased from *m*- to *o*-KNbO_3_ NWs, suggesting that the O chemical environment is different in the *m*- and *o*-KNbO_3_ NWs. High-resolution X-ray photoelectron spectroscopy (XPS) spectra (Fig. 2c) indicate that Nb 3d_5/2_ is *ca.* 206.5 and 206.6 eV for *m*- and *o*-KNbO_3_ NWs, respectively. Fig. 2d reveals that the *m*- and *o*-KNbO_3_ NWs present similar valence band (VB) maxima (*ca.* 2.2 eV) and line shape, and no detectable oxygen vacancy defects exist. Since the *o*-KNbO_3_ NWs have a greater bandgap than *m*-KNbO_3_ NWs, the conduction band (CB) minimum of the *o*-KNbO_3_ NWs should be raised with respect to that of *m*-KNbO_3_ NWs. The above results demonstrate the intrinsic differences of the geometric structure and electronic structure between the *m*- and *o*-KNbO_3_ NWs, albeit that they share similar size, crystallinity and specific surface area.

**Fig. 3:**
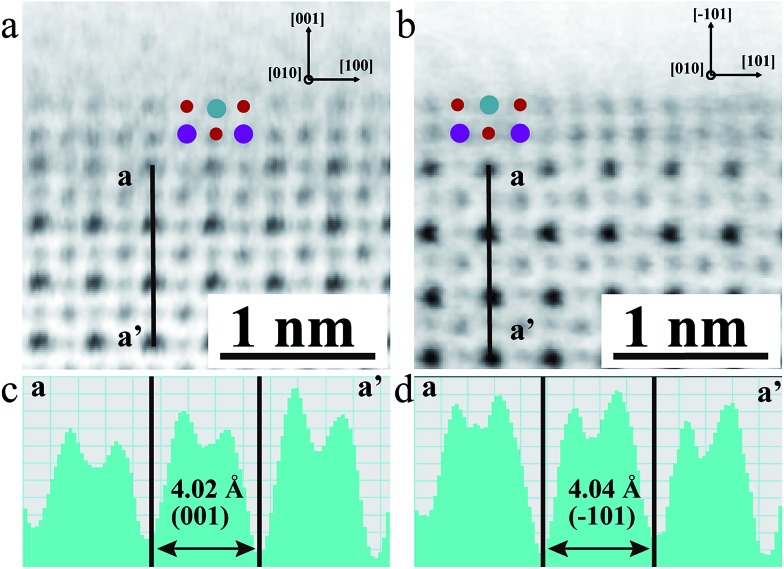
Representative atomic-scale surface structure of *m*- and *o*-KNbO_3_ NWs by ABF-STEM profile-view imaging. (a) *m*-KNbO_3_ viewed along the [010] direction (cyan: NbO; red: O; purple: K). (b) *o*-KNbO_3_ viewed along the [010] direction (cyan: NbO; red: O; purple: K). (c) Corresponding line profiles showing the image intensity as a function of the position in image (a) along a–a′. (d) Corresponding line profiles showing the image intensity as a function of the position in image (b) along a–a′.

To examine the geometric structure of the *m*- and *o*-KNbO_3_ NWs at an atomic level, we utilized annular-bright-field (ABF)-STEM.^49^ In general, the contrast of ABF imaging depends on *Z*^1/3^ (*Z* is the atomic number) and light elements like O can be directly visualized at a subangstrom resolution.^50^ Therefore, the understanding of ABF imaging contrast in metal oxides is straightforward. Herein, the spots of black, dark grey and light grey correspond to NbO (*Z*_Nb_ = 41), K (*Z*_K_ = 19) and O (*Z*_O_ = 8) columns seen end-on, respectively. The representative surface structure of the *m*- and *o*-KNbO_3_ NWs is revealed by profile-view imaging (Fig. 3a and b) taken along the [010] direction. Cyan, red and purple symbols denote NbO, K and O columns, respectively. According to the corresponding line profiles (Fig. 3c and d), the spacing between NbO columns (4.02 Å and 4.04 Å) fits well to *m*-KNbO_3_ (001) and *o*-KNbO_3_ (101), respectively. The outmost surface layer of *m*-KNbO_3_ (001) and *o*-KNbO_3_ (101) is NbO_2_ terminated, where the exposed Nb cation (denoted as Nb_5c_) is bonded to five oxygen anions. In general, the coordinatively unsaturated surface cations often act as active sites in heterogeneous photocatalysis.^32^ Additionally, neither surface relaxations nor reconstructions are observed. Other facets like {100} and {010} of *m*-KNbO_3_, and {010} of *o*-KNbO_3_, are also predominantly NbO_2_ terminated (data not shown).

**Fig. 4:**
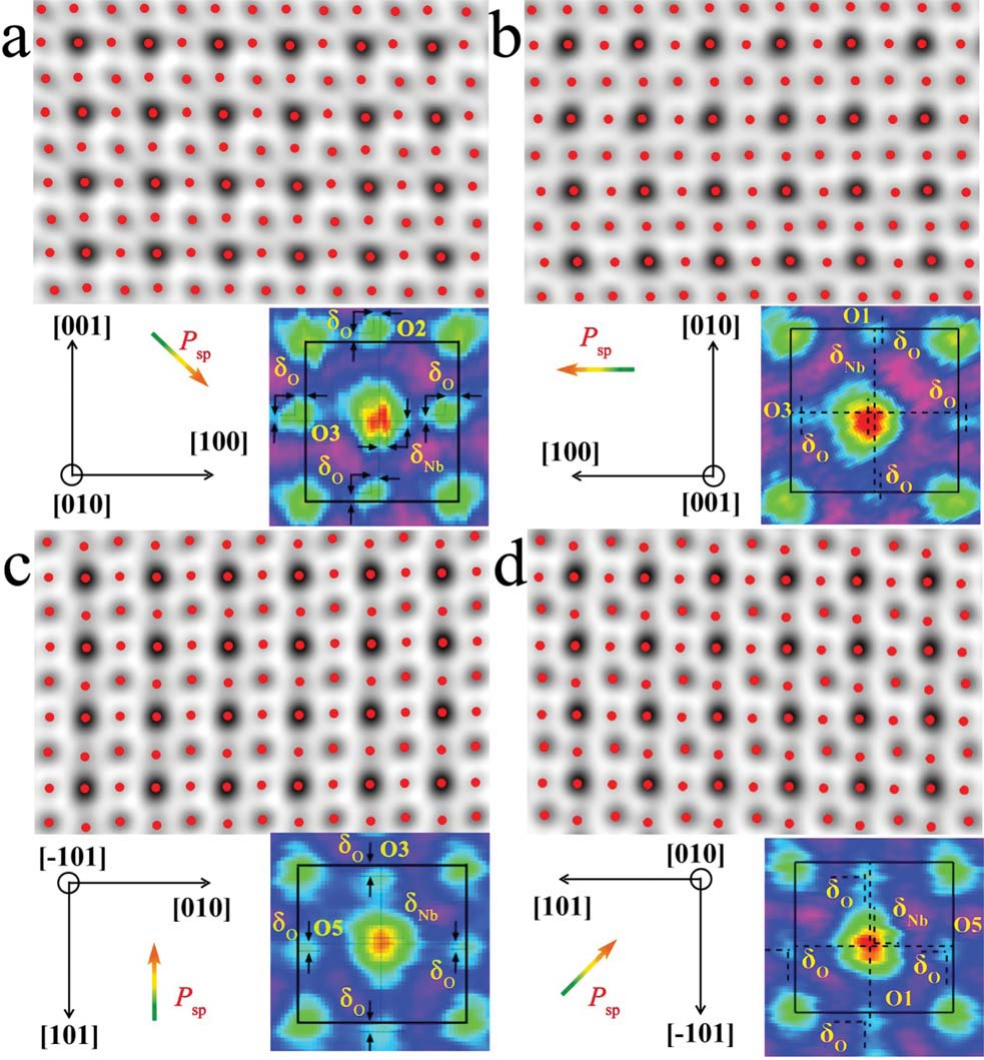
Representative atomic-scale bulk structure of the *m*- and *o*-KNbO_3_ NWs by ABF-STEM imaging. The images are overlaid with red dots that represent atomic column positions at an accuracy of several picometers obtained by Peak Pairs Analysis. Corresponding zoom-in colour-enhanced ABF images are highlighted. (a) *m*-KNbO_3_ NWs viewed along the [010] direction. (b) *m*-KNbO_3_ NWs viewed along the [001] direction. (c) *o*-KNbO_3_ NWs viewed along the [101] direction. (d) *o*-KNbO_3_ NWs viewed along the [010] direction. *P*_sp_ is the polarization.

The local bulk structure of the KNbO_3_ NWs is directly visualized as well. To identify the precise atomic positions from ABF images at an accuracy of several picometers and then quantitatively determine the delicate structural difference, we employed Bragg filtering and the “Find Peaks” option based on Peak Pairs Analysis.^51^,^52^ The atomic column locations are obtained as coordinates (*x*, *y*) by fitting a two-dimensional (2D) quadratic function and calculating the maxima of the atomic column positions. The ABF imaging of the *m*- and *o*-KNbO_3_ NWs viewed along different crystallographic directions with overlaid red dots obtained by Peak Pairs Analysis in Fig. 4 clearly shows the precise atomic column positions. As schematically illustrated by representative zoom-in colour-enhanced ABF images (Fig. 4), clear atomic displacements with respect to the KNbO_3_ cubic structure are observed.^44^ For example, the zoom-in colour-enhanced ABF images in Fig. 4a show that the NbO columns shift to the lower-right and the O columns shift to the upper-left within the rectangles formed by four K columns. Table S1† displays the displacements of the Nb atoms and O atoms along different directions, denoted as *δ*_Nb_ and *δ*_O_, respectively. Herein, different O atoms are numbered according to the coordinates. The atomic displacements in the *m*-KNbO_3_ NWs of O1, O2, O3 and Nb1 are –0.12 ± 0.05, –0.11 ± 0.03, –0.10 ± 0.04 and 0.05 ± 0.03 Å along the [100] direction, and 0.11 ± 0.06, 0.10 ± 0.04, 0.11 ± 0.03 and –0.05 ± 0.04 Å along the [001] direction. No detectable displacements are probed for the Nb and O atoms along the [010] direction. As for the *o*-KNbO_3_ NWs, the atomic displacements of O1, O3, O5 and Nb1 are 0.22 ± 0.03, 0.21 ± 0.05, 0.21 ± 0.04 and –0.15 ± 0.05 Å along the [101] direction, and 0.21 ± 0.06, 0.20 ± 0.03, 0.21 ± 0.05 and –0.14 ± 0.04 Å along the [101] direction. Additionally, no detectable displacements are observed along the [010] direction for the Nb and O atoms. The observed difference in atomic displacements for *m*- and *o*-KNbO_3_ is due to the intrinsic structural discrepancy. The atomic displacements are corroborated by spin-polarized DFT calculations as shown in Tables S2 and S3,† which are in good agreement with the STEM measurements.

In the KNbO_3_ unit cell, spontaneous ferroelectric polarization (denoted as *P*_sp_) arises from the displacement of the positive charge center and negative charge center. K contribution to the total polarization is negligible due to the essential ionic interaction between K and O.^53^*P*_sp_ is calculated on the basis of lattice parameters, atomic displacements and Born effective charges of the ions, while the Born effective charges are determined using spin-polarized DFT calculations.^44^,^54^ The results are shown in Tables S2 and S3.† The vector of *P*_sp_ is pointed from the net negative to the net positive charge. As for *m*-KNbO_3_, the local polarization is 20 μC cm^–2^ along the 101 direction. In the case of *o*-KNbO_3_, the local polarization is 42 μC cm^–2^ along the 001 direction. Due to the crystal size limitations, mapping polarization domains in KNbO_3_ NWs by STEM imaging is difficult. Nevertheless, the delicate structural variation-derived local polarization is distinct between *m*- and *o*-KNbO_3_ NWs and expected to cause different photocatalysis.

**Fig. 5:**
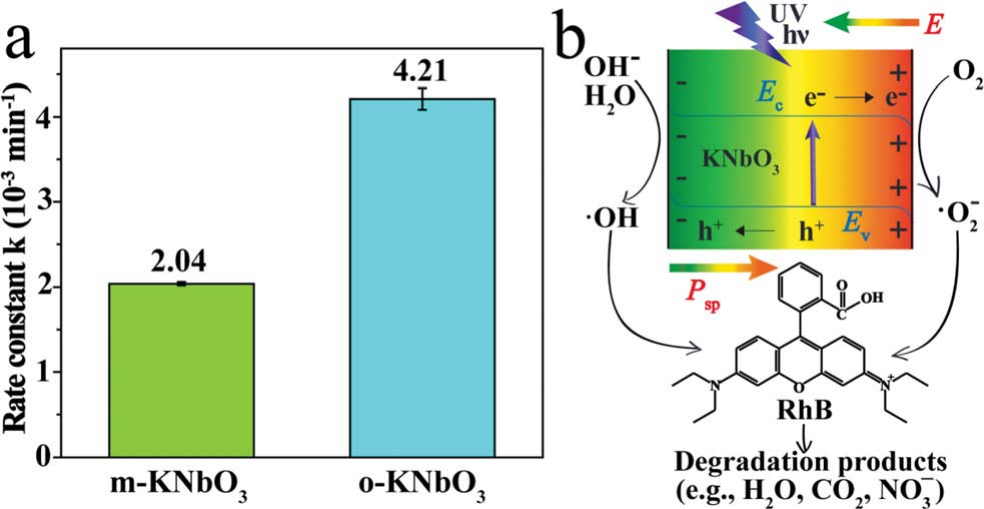
(a) Photoreactivity of the *m*- and *o*-KNbO_3_ NWs towards RhB degradation in water under UV light. (b) Representative schematic illustration of charge separation in the KNbO_3_ NWs under UV light and associated photocatalytic degradation of RhB. *E*_v_ is the valence band edge, *E*_c_ is the conduction band edge, *P*_sp_ is the polarization and *E* is the internal electric field.

The photoreactivity of the KNbO_3_ NWs was assessed towards RhB degradation in water under UV light. The reaction rate constants were calculated and the results are displayed in Fig. 5a. In the absence of KNbO_3_, RhB degradation is negligible (data not shown). Under identical experimental conditions, the *o*-KNbO_3_ NWs displayed photoreactivity (*k* = 4.21 × 10^–3^ min^–1^) that is about two-fold as large as that of the *m*-KNbO_3_ NWs (*k* = 2.04 × 10^–3^ min^–1^). The physicochemical properties of the as-prepared KNbO_3_ NWs are summarized in Table 1. Furthermore, the photostability was examined and the results are shown in the ESI (Fig. S3–S5†). With regard to the reaction products concerning RhB degradation, such as total organic carbon (TOC), intermediate products, and inorganic mineralization species, detailed analyses along with proposed reaction pathways are given in the ESI (Tables S4, S5 and Fig. S6–S8).†

**Table 1:** Physicochemical properties of *m*- and *o*-KNbO_3_ NWs

Samples	Surface area [m^2^ g^–1^]	Bandgap [eV]	Exposed facets	Polarization [μC cm^–2^]	Density of Nb_5c_ [atoms nm^–2^]	Reaction rate [ ×10^–3^ min^–1^]
*m*-KNbO_3_	4.7	3.15	{010}	20	5.90	2.04
			{001}		5.92	
			{100}		5.93	
*o*-KNbO_3_	4.8	3.25	{010}	42	5.90	4.21
			{101}		6.14	

To explore the photoreactivity difference between the *m*- and *o*-KNbO_3_ NWs, a brief overview of photocatalytic process follows. In general, semiconductor-based photocatalysis involves three steps: the photogeneration of e^–^–h^+^ pairs, the separation and transport of e^–^–h^+^, and their reaching the surface and reaction with the adsorbates. In the first step, the electronic structure, like the bandgap of a given photocatalyst, determines the light absorption and redox potentials of photo-induced charge carriers.^31^ Because the *o*-KNbO_3_ NWs have a greater bandgap than the *m*-KNbO_3_ NWs, the *o*-KNbO_3_ NWs possess a higher CB minimum than the *m*-KNbO_3_ NWs and thus generate more strongly reductive electrons in photocatalysis.^34^ The discrepancy of electronic structure is in good agreement with structural variations between *m*- and *o*-KNbO_3_. The key issue in the second step is the charge pair recombination. Utilizing an internal electric field as a driving force is an emerging approach to suppress e^–^–h^+^ recombination.^18^,^19^ Recent photocatalytic studies on the effects of an internal electric field in BaTiO_3_,^55^ ZnS,^56^ AgI^4^ and BiVO_4_ 57 were only qualitatively discussed. In the present study, the magnitude of local polarization for *m*- and *o*-KNbO_3_ is determined. The *o*-KNbO_3_ NWs present polarization of 42 μC cm^–2^ along the 001 direction. As for *m*-KNbO_3_, the polarization is 20 μC cm^–2^ along the 101 direction. As such, a positive charge on the surface is produced when polarization points from the bulk to the surface and *vice versa*. To compensate this bound charge, both internal screening and external screening occur.^55^ For internal screening, the free charge carriers in the bulk move to the opposite charged surfaces, resulting in respective downward and upward band bending on positive and negative charged surfaces as shown in Fig. 5b. The resulting band bending further provides a driving force for the spatial separation of photo-excited electrons and holes.^19^ External screening refers to the adsorption of molecules like RhB. Based on the above analysis, the *o*-KNbO_3_ NWs exhibit more enhanced photoreactivity than the *m*-KNbO_3_ NWs, illustrating that the greater the local polarization, the more enhanced the photoreactivity. The third step is relevant to the photocatalyst surface structure. Herein, the respective density of Nb_5c_ for *m*-KNbO_3_ NWs {010}, {001} and {100} is 5.90, 5.92 and 5.93 atoms nm^–2^, very similar to 5.90 and 6.14 atoms nm^–2^ for {010} and {101} of the *o*-KNbO_3_ NWs. Therefore, in this work the surface structure is most likely not a leading factor in causing the distinct photocatalytic performance. Considering that the *o*-KNbO_3_ NWs are able to generate more strongly reductive electrons than their *m*-KNbO_3_ counterparts under UV light, we can conclude that both ferroelectric polarization and electronic structure synergistically dictate the photocatalytic performance. Unlike previous studies that emphasized the effects of exposed facets^34^–^38^ in photocatalysis, our results indicate that ferroelectric polarization dictates the photoreactivity by driving e^–^ and h^+^ to the photocatalyst surface to trigger the photocatalytic reaction, albeit the facets of the *m*- and *o*-KNbO_3_ NWs expose comparable surface low-coordinate Nb density. This is, therefore, a unique route for directing the transport of photo-excited charge carriers and enhancing the photoreactivity.

## Conclusions

In summary, with a combination of advanced ABF-STEM imaging and DFT calculations, we directly probed the atomic surface structure, measured delicate atomic displacements at an accuracy of several picometers, and quantified associated local polarization in single-crystalline *m*- and *o*-KNbO_3_ NWs with comparable size, crystallinity and specific surface area. Orthorhombic KNbO_3_ nanowires displayed RhB photodegradation about two-fold as large as their monoclinic counterparts and the underlying mechanism can be rationalized as a novel synergy of delicate atomic structural variation-derived ferroelectric polarization and electronic structure. Additionally, RhB degradation pathways are proposed, with an emphasis on *N*-deethylation and conjugated structure cleavage. Our results are potentially applicable to a range of perovskite ferroelectric materials functional in light-mediated environment remediation and energy production.

## Supplementary Material

Supplementary information
