# Supplementary material for: Insights into the structure–photoreactivity relationships in well-defined perovskite ferroelectric KNbO3 nanowires
Source: Chem Sci. 2015 Apr 23;6(7):4118–23. doi: 10.1039/c5sc00766f (PMC5707469; doi:10.1039/c5sc00766f)
Supplement: Supplementary file 1 [file SC-006-C5SC00766F-s001.pdf]

Electronic Supplementary Material (ESI) for Chemical Science.
This journal is © The Royal Society of Chemistry 2015

Electronic Supplementary Information

Insights into Structure-Photoreactivity Relationships in Well-Defined Perovskite Ferroelectric $KNbO_3$ Nanowires

Tingting Zhang, Wanying Lei, Ping Liu, José A. Rodriguez, Jiaguo Yu, Yang Qi, Gang Liu and Minghua Liu**

Experimental Section

Material synthesis: All chemicals (ACS grade) were purchased from Alfa Aesar and used without further purification. When applicable, Milli-Q water (18 MΩ·cm in resistivity, Millipore Corporation) was used. m-$KNbO_3$ NWs were hydrothermally synthesized using a method adapted from prior work.[1] Briefly, 12.6 g potassium hydroxide (KOH, 85% min, K_2CO_3 2.0% max) was completely dissolved into 15 mL Milli-Q water under magnetic stirring. Then, 0.874 g metallic Nb powder (325 mesh, 99.8%) was added. The mixture was stirred vigorously at room temperature for 30 min and transferred into a Teflon-lined stainless steel autoclave (50 mL) and heated at 160 ^{o}C for 12 h. After cooling down to room temperature, a white precipitate was collected and washed several times with Milli-Q water and ethanol. The as-obtained sample was dried at 60 ^{o}C for 12 h. o-$KNbO_3$ NWs was synthesized via a hydrothermal process.[2] Briefly, 18 g potassium hydroxide (KOH, 85% min, K_2CO_3 2.0% max) was dissolved into 20 mL Milli-Q water under vigorously stirring and 0.5 g Nb_2O_5 (puratronic, 99.9985%) was slowly added. Then, 0.7 g surfactant sodium dodecyl sulfate (SDS, 99%) was mixed. The mixture was transferred into a Teflon-lined stainless steel autoclave (100 mL) and heated to 180 ^{o}C for 48 h. After cooling down to room temperature, the as-obtained white precipitate was washed with Milli-Q water and ethanol many times, and then dried at 60 ^{o}C for 12 h.

Characterization: The crystal structures of the as-prepared samples were determined by a Rigaku Corporation XRD (XRD-6000) with Cu Kα radiation (λ=0.154178 nm, 50 kV, 300 mA) at a scanning rate of 10° min^{-1} and 0.2° min^{-1} in the 2θ range of 10−85° and 44−46°, respectively. The sample morphology was characterized using a Hitachi S4800 cold field-emission SEM. HRTEM images were obtained using Tecnai G^2 F20 U-TWIN transmission electron microscope operating at an accelerating voltage of 200 kV. The BET specific surface area was measured using a Micromeritics ASAP 2000. Tapping-mode AFM measurements were carried out under ambient conditions using a Dimension 3100 (Veeco). XPS data were collected using an ESCALab 250 Xi electron spectrometer from Thermo Scientific Corporation. Monochromatic 150 W Al Kα radiation was utilized and low-energy electrons were used for charge compensation to neutralize the samples. The binding energies were referenced to the adventitious C 1s line at 284.8 eV. The O *K*-edge XANES spectra were collected at the Photoelectron Spectroscopy Station of the Beijing Synchrotron Radiation Facility of Institute of High Energy

Physics, Chinese Academy of Sciences. The Photoelectron Spectroscopy Station was equipped with an ultrahigh vacuum (UHV) chamber with a base pressure of ~8×10^{-10} Torr. All O *K*-edge spectra were recorded at room temperature with an overall energy resolution of 0.5 eV in the total electron yield (TEY) detection mode. The spectra were normalized to the incident photon flux and the energy calibration was performed with respect to an Au foil. DRUV-Vis spectra were obtained in the range of 200−800 nm using Varian Cary 5000 with MgO as a reference. Aberration-corrected ABF-STEM imaging was performed using a JEOL JEM ARM200F (Tokyo, Japan) TEM equipped with two CEOS (Heidelberg, Germany) probe aberration correctors. The attainable spatial resolution of the microscope is 90 pm at an incident angle of 25 mrad. The finite pixel size of ABF-STEM images is 0.10 Å. Measurements of the atomic positions were carried out in the STEM images at an accuracy of several picometers using Bragg filtering and ‘‘Find Peaks’’ option (Peak Pairs Analysis software package by HREM Research).[3] The products of post-reactions were evaluated using a series of analytical instruments. TOC was detected on a Teledyne Tekmar TOC Fusion Analyzer. During the TOC analysis, inorganic carbon ions were removed using acid treatment before organic carbon oxidized by a powerful UV reaction chamber to form CO_2 which is evacuated by a CO_2 gas detector. The concentrations of mineralization species nitrate ions (NO_3^-) were probed on an ion chromatography (ICS-1500, Dionex) with an UVD-500 UV/VIS Detector, and the current was set as 45 mA. The eluent consists of sodium carbonate (9 mmol·L^{-1}) and the flow rate is 1.00 mL·min^{-1}. The volume of analyzed samples is 200 μL and the cell temperature is maintained at 35 °C. The intermediates of degraded RhB were monitored on Waters Acquity Ultra Performance Liquid Chromatography (UPLC) H-Class in line with a Waters Xevo G2-S QTof mass spectrometer (MS) equipped with a C18 column (ACQUITY UPLC BEH C18 1.7 μm 2.1×150 mm). The system was operated in a positive ion mode (electrospray ionization, ESI). 5.00 μL of aliquots was injected for analysis. The eluent contains water and acetonitrile, with column temperature of 35 °C and flow rate of 0.3 mL·min^{-1}. The source parameters were listed as following: capillary voltage 3.50 kV, sampling cone voltage 30.0 V, source offset 350 V, source temperature 100 °C, desolvation temperature 250 °C, cone gas flow 50 L·h^{-1} and desolvation gas flow 600 L·h^{-1}.

Photocatalytic degradation of RhB: The photocatalytic activities of the as-prepared samples were evaluated toward the degradation of RhB (Alfa Aesar) in water. Briefly, 50 mg $KNbO_3$ was

mixed with 50 mL RhB (8×10^{-6} M) in a 150 mL Pyrex flask. The mixture was sonicated in cool water for 10 min. Before irradiation, the suspension was magnetically stirred in dark to establish a complete adsorption-desorption equilibrium. A 300 W xenon lamp was located at ca. 10 cm away from the center of the flask. The irradiation intensity of UV (λ: 250−380 nm) in the center of the flask was measured to be 290 $mW\cdot cm^{-2}$ by using a Newport optical meter (842-PE). During the irradiation, the reaction suspension with initial pH of 6.1 was magnetically stirred at room temperature. About 3.5 mL aliquots were sampled at certain time intervals, followed by centrifugation at 15000 rpm for 5 min to remove catalyst particulates. The optical absorption intensity at 554 nm of the obtained dye solution was measured to determine the RhB concentration by using PerkinElmer Lambda 950 UV-vis spectrophotometer. In case of stability test, for each cycle a fresh dye solution was used and after test the catalyst was thoroughly washed with water and ethanol by centrifugation for several times, and dried at 60 °C.

Computational methods: The spin-polarized DFT calculations were performed using the CASTEP (Cambridge Serial Total Energy Package) suite of programs.[4] The Kohn-Sham one-electron equations were solved on a basis set of plane waves with energy cutoff of 1200 eV and norm-conserving were used to describe the ionic cores.[5] Brillouin Zone integration was approximated by a sum over special k-points selected using the Monkhorst-Pack scheme.[6] Enough K-points (8×8×8) were chosen to make sure that there was no significant change in the calculated energies when a larger number of K points were used. The exchange-correlation energy and the potential were described by the Wu-Cohen (WC) functional.[7] The calculations start from the atomic coordinates and unit cells determined from diffraction data of m- and o-$KNbO_3$.[1, 8] The calculations are sufficiently converged to allow the atomic structures to be optimized until the residual forces on the atoms are less than 0.005 $eV\cdot Å^{-1}$. In the calculations of Born effective charge, density functional perturbation theory (DFPT)[9] was used, where the core charge of the ion and the corresponding component of the response of all the shells present to an atomic displacement.

Results

To investigate the photostability of m- and o-$KNbO_3$ NWs, the recycling test was performed and the results are shown in Fig. S3†. After three cycles of degradation of RhB solution under

UV-light, no significant loss of activity of m- and o-$KNbO_3$ NWs was observed. Our results demonstrate that both m- and o-$KNbO_3$ NWs present high stability during the photocatalytic reactions and negligible photocorrosion occurs. Fig. S4 and S5† show the XRD patterns and SEM images of m- and o-$KNbO_3$ NWs after the photocatalytic reactions. No significant structural and morphological changes were observed.

TOC is the most relevant parameter for the determination of all organically bound carbon atoms in dissolved and undissolved organic compounds in the samples. Table S4† shows the TOC before and after the reactions. The TOC change is 0.342 and 1.586 ppm in the presence of m- and o-$KNbO_3$ NWs, respectively. On the other hand, the change of NO_3^- concentrations (Table S4†) that in part reflect the mineralization degree of degraded RhB is also higher in the presence of o-$KNbO_3$ than that of m-$KNbO_3$ NWs. Results of both TOC and NO_3^- measurements are in agreement with the reaction rate constants as seen in Table 1, indicating that the photoreactivity of o-$KNbO_3$ is greater than m-$KNbO_3$ NWs.

Major products generated for RhB degradation under UV in the presence of $KNbO_3$ photocatalysts were probed by UPLC-MS and the results are displayed in Table S5†. A series of intermediates are identified by using the m/z values in the mass spectra, including m/z 283 (compound A, retention time 2.79 min), m/z 339 (compound B retention time 2.99 min), m/z 395 (compound C retention time 3.14 min), m/z 216 (compound D retention time 3.52 min), m/z 260 (compound E retention time 3.65 min), m/z 244 (compound F retention time 4.61 min), m/z 289 (compound G retention time 4.68 min), m/z 415 (compound H retention time 5.32 min), m/z 273 (compound I retention time 5.54 min), and m/z 317 (compound J retention time 5.60 min). As seen from Fig. S6 and S7†, before light illumination, there is a pronounced peak at 5.85 min (m/z 443 corresponding to RhB, denoted as compound K). In the presence of m-$KNbO_3$ NWs (Fig. S6†), the signal for RhB is still present after 210 min irradiation, though the intensity of RhB signal is decreased gradually. In contrast, for o-$KNbO_3$ (Fig. S7†) the signal for RhB is absent in 120 min. It is notable that the concentration of the intermediates is enhanced with irradiation time.

The primary reaction intermediates of RhB degradation catalyzed by a variety of photocatalysts under light were reported previously, focusing on byproducts with m/z either larger than 300 by N-deethylation[10] or smaller than 200[11] due to benzene removal and

opening-ring rapture. In general, in photocatalytic RhB degradation, there is stepwise loss of ethyl groups and the subsequent formation of five N-deethylation products (i.e., m/z = 415, m/z = 387, m/z = 387, m/z = 359 and m/z = 331 with absorption maximum located at 541, 525, 529, 514 and 502 nm, respectively).[10] Based on the analysis of major products in the current study, in particular intermediates with m/z in the range of 200−300 (i.e., m/z = 216, 273 and 289) that are revealed for the first time, we tentatively propose new photocatalytic degradation pathways of RhB as shown in Fig. S8†, involving N-deethylation as well as the cleavage of the conjugated structure. In view of total ion chromatography of RhB, there is only one N-deethylation product (m/z = 415), suggesting that N-deethylation process is dominated by a simultaneous loss of the ethyl groups rather than a stepwise process as reported in previous studies. Considering the less amounts of N-deethylation intermediates, it is reasonable to conclude that cleavage is the major reaction route compared with N-deethylation. The total ion chromatography of RhB in the presence of m- and o-$KNbO_3$ NWs is very similar, implying the same degradation pathways as shown in Fig. S8†. The degradation pathways depicted in Fig. S8† include mainly two parts. On the right panel of Fig. S8†, N-deethylated intermediate (m/z = 415) were degraded into a possible intermediate corresponding to an m/z value of 317 and further degraded into intermediates with m/z values of 289, 273, 260 and 244. On the left panel of Fig. S8†, the intermediate with an m/z value of 395 might be further degraded into the intermediates with m/z values of 339, 283 and 216. Then the molecules were mineralized to CO_2, H_2O and NO_3^-, which are proved by TOC and ion chromatography measurements. In conclusion, our results demonstrate that UV induced RhB photodegradation in water by m- and o-$KNbO_3$ NWs involves two processes, i.e., N-deethylation and conjugated structure cleavage, with cleavage as a major reaction route. Both processes occur simultaneously and decompose RhB to colorless compounds and ultimately to CO_2, H_2O and NO_3^-.

Table S1. The shifts of Nb and O atoms along different directions based on statistical ABF-STEM imaging measurements. The unit is Å.

Atom	m-$KNbO_3$			Atom	o-$KNbO_3$		
	[100]	[010]	[001]		[−101]	[010]	[101]
O1	−0.12±0.05	−	0.11±0.06	O1	0.22±0.03	−	0.21±0.06
O2	−0.11±0.03	−	0.10±0.04	O3	0.21±0.05	−	0.20±0.03
O3	−0.10±0.04	−	0.11±0.03	O5	0.21±0.04	−	0.21±0.05
Nb1	0.05±0.03	−	−0.05±0.04	Nb1	−0.15±0.05	−	−0.14±0.04

Table S2. Calculated coordinates of atoms, atomic displacement and Born effective charge tensor of m-$KNbO_3$.

Atom	Coordinates of atoms			Atomic displacement [Å]			Born effective charge		
	x	y	z	x	y	z	Z^*_{xx}	Z^*_{yy}	Z^*_{zz}
O1	−0.621	0.000	0.403	−0.08	0	0.07	−1.632	−8.094	−1.654
O2	0.382	0.500	0.900	−0.07	0	0.05	−1.618	−1.758	−7.160
O3	−0.116	0.500	0.400	−0.06	0	0.06	−7.035	−1.746	−1.629
K1	−0.102	0.000	−0.114				1.129	1.121	1.126
Nb1	0.406	0.500	0.378	0.03	0	−0.03	9.157	10.48	9.316

Table S3. Calculated coordinates of atoms, atomic displacement and Born effective charge tensor of o-$KNbO_3$.

Atom	Coordinates of atoms			Atomic displacement [Å]			Born effective charge		
	x	y	z	x	y	z	Z^*_{xx}	Z^*_{yy}	Z^*_{zz}
O1	0.000	0.500	0.032	0	0	0.11	−1.638	−8.105	−1.660
O2	0.500	0.500	0.532	0	0	0.11	−1.638	−8.105	−1.660
O3	0.249	0.000	0.278	0.03	0	0.09	−4.519	−1.766	−4.096
O4	0.749	0.000	0.778	0.03	0	0.09	−4.519	−1.766	−4.096
O5	−0.249	0.500	0.278	−0.03	0	0.09	−4.519	−1.766	−4.096
O6	0.251	0.000	0.778	−0.03	0	0.09	−4.519	−1.766	−4.096
K1	0.000	0.500	0.513				1.137	1.120	1.115
K2	0.500	0.500	1.013				1.137	1.120	1.115
Nb1	0.000	0.000	0.006	0	0	−0.09	9.539	10.516	8.737
Nb2	0.500	0.000	0.506	0	0	−0.09	9.539	10.516	8.737

Table S4. The change of TOC, NO_3^- concentrations in RhB solution before and after photocatalytic reactions in the presence of $KNbO_3$ under UV.

Samples	TOC [ppm]			NO_3^- [ppm]		
	Before reaction	After reaction	Change of TOC	Before reaction	After reaction	Change of NO_3^-
m-$KNbO_3$	11.052	10.710	0.342	0.01526	0.8653	0.8500
o-$KNbO_3$	11.052	9.466	1.586	0.01526	1.4019	1.3866

Table S5. Identification of major intermediate products derived from RhB degradation by UPLC-MS (positive ion mode ESI mass spectra) in the presence of $KNbO_3$ NWs under UV.

Peak	Retention time [min]	m/z	Formula
A	2.79	283	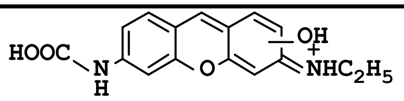
B	2.99	339	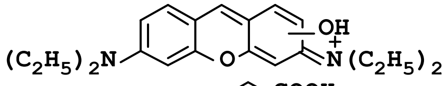
C	3.14	395	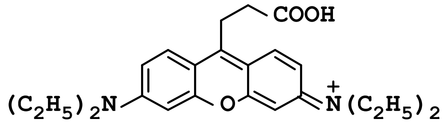
D	3.52	216	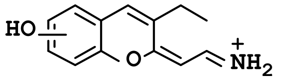
E	3.65	260	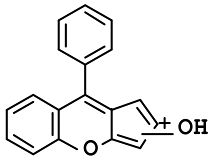
F	4.61	244	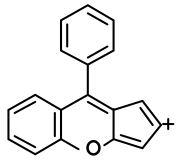
G	4.68	289	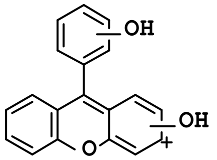
H	5.32	415	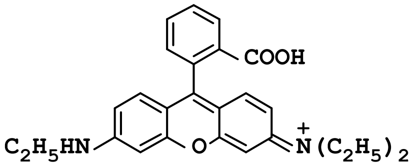
I	5.54	273	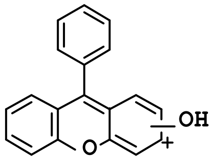
J	5.60	317	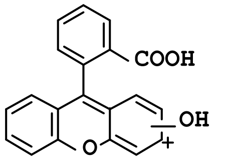
K	5.86	443	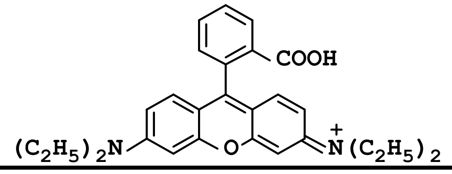

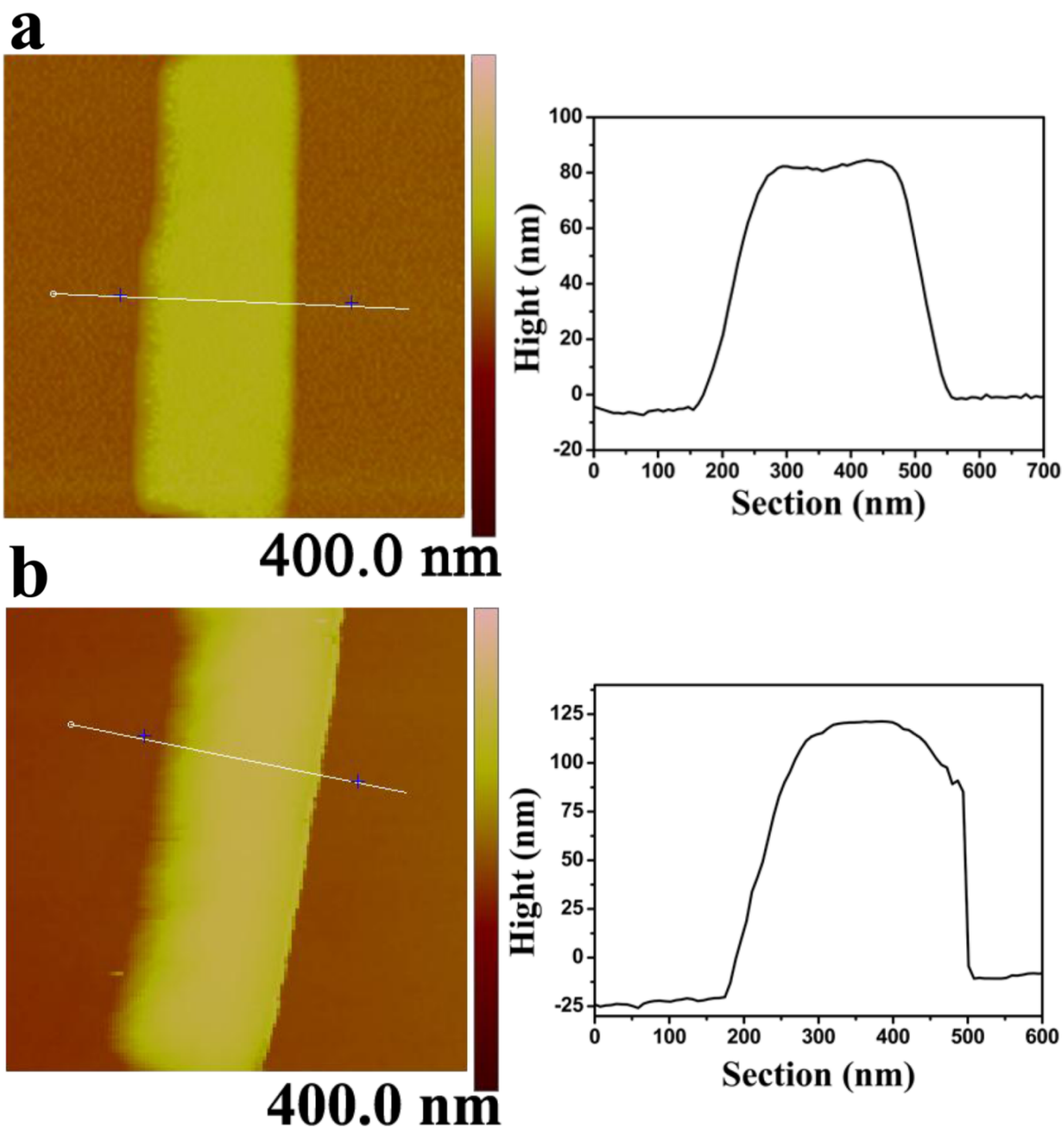

Fig. S1. Representative tapping mode AFM topographical images (a) (972.8×972.8) nm of m-$KNbO_3$ NWs and (b) (895.8×895.8) nm of o-$KNbO_3$ NWs deposited on a silicon wafer. The cross-sectional views correspond to the lines drawn in the images.

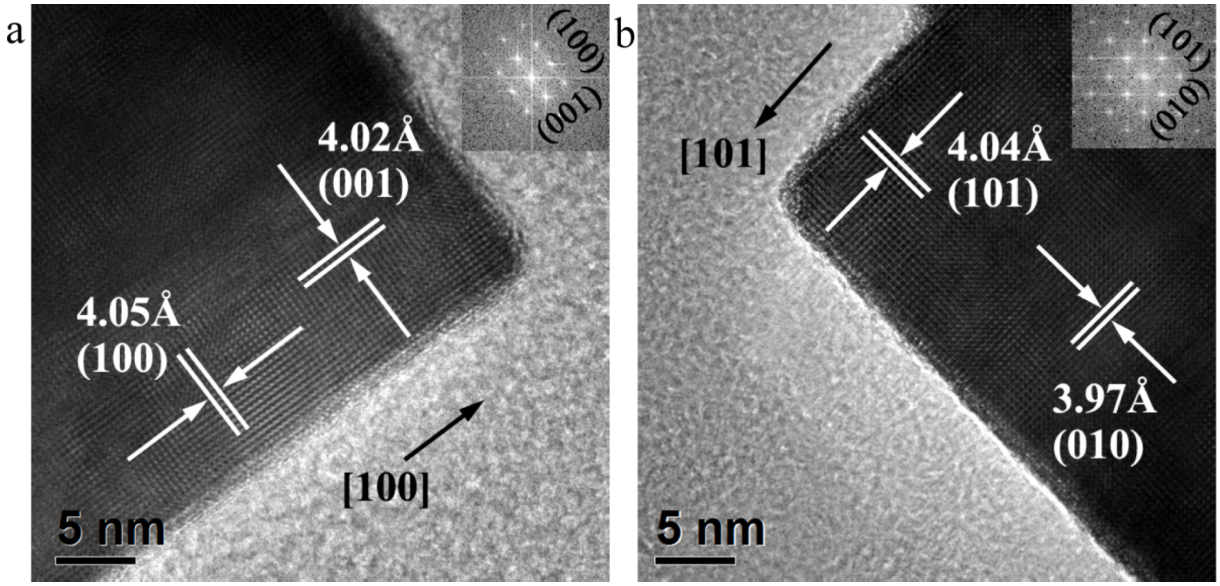

Fig. S2. (a) HRTEM image of a typical m-$KNbO_3$ NW. (b) HRTEM image of a typical o-$KNbO_3$ NW. Inset: Fast Fourier Transform (FFT) patterns.

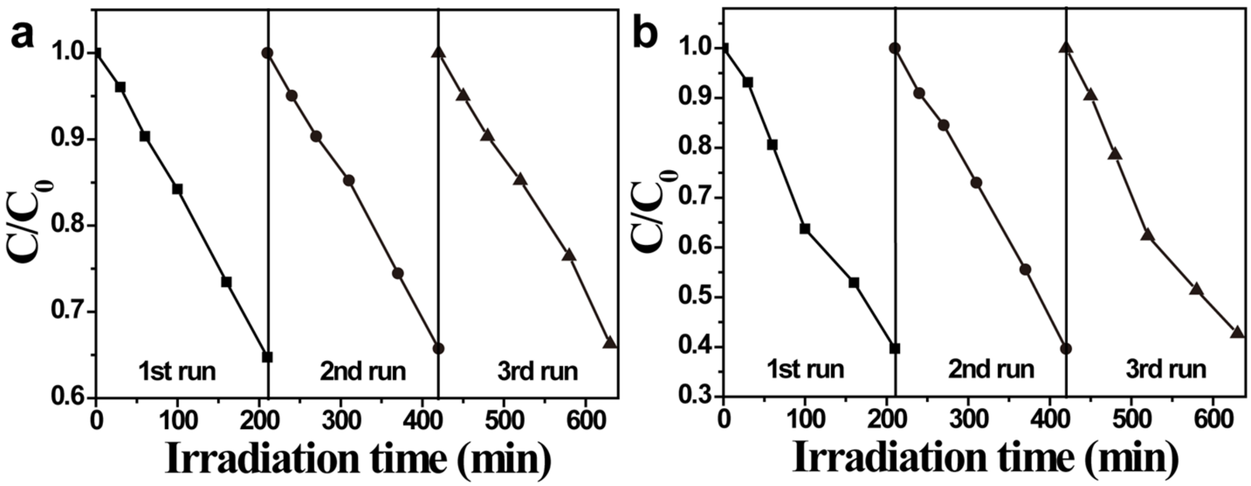

Fig. S3. Recycling test of (a) m-$KNbO_3$ and (b) o-$KNbO_3$ NWs in the photocatalytic degradation of RhB under UV-light. The duration of light exposure in each cycle is 210 min.

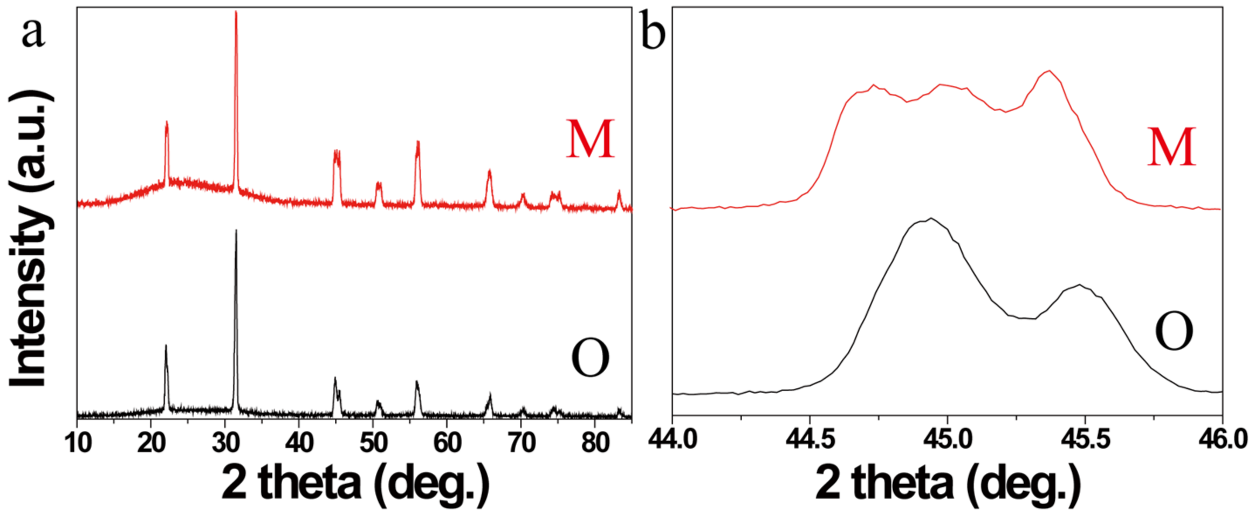

Fig. S4. (a) Powder XRD patterns of m- and o-$KNbO_3$ NWs after the photocatalytic reactions. (b) High-resolution XRD patterns in the range of 44°−46°. M: m-$KNbO_3$ NWs; O: o-$KNbO_3$ NWs.

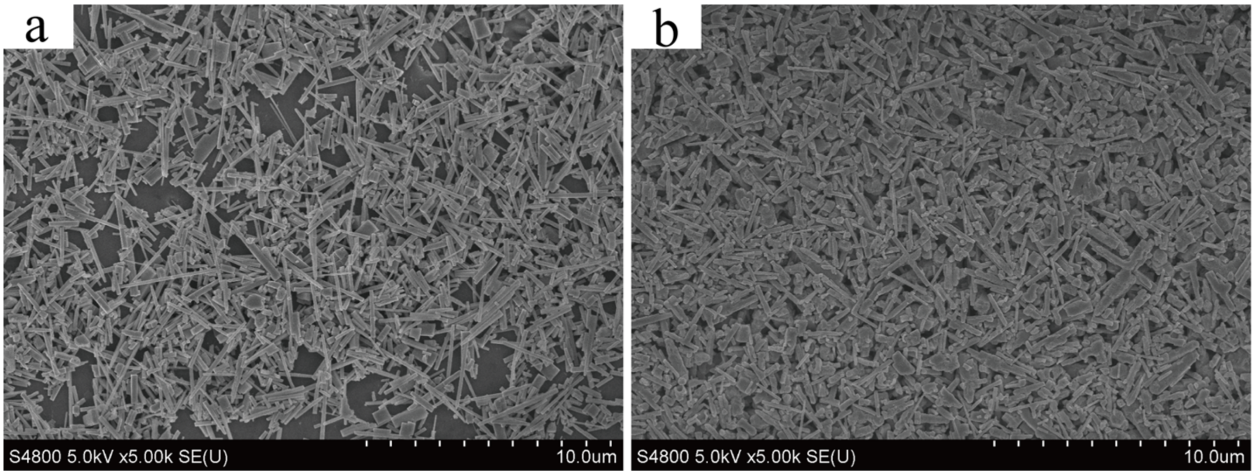

Fig. S5. SEM image of $KNbO_3$ NWs after the photocatalytic reactions. (a) m-$KNbO_3$ NWs. (b) o-$KNbO_3$ NWs.

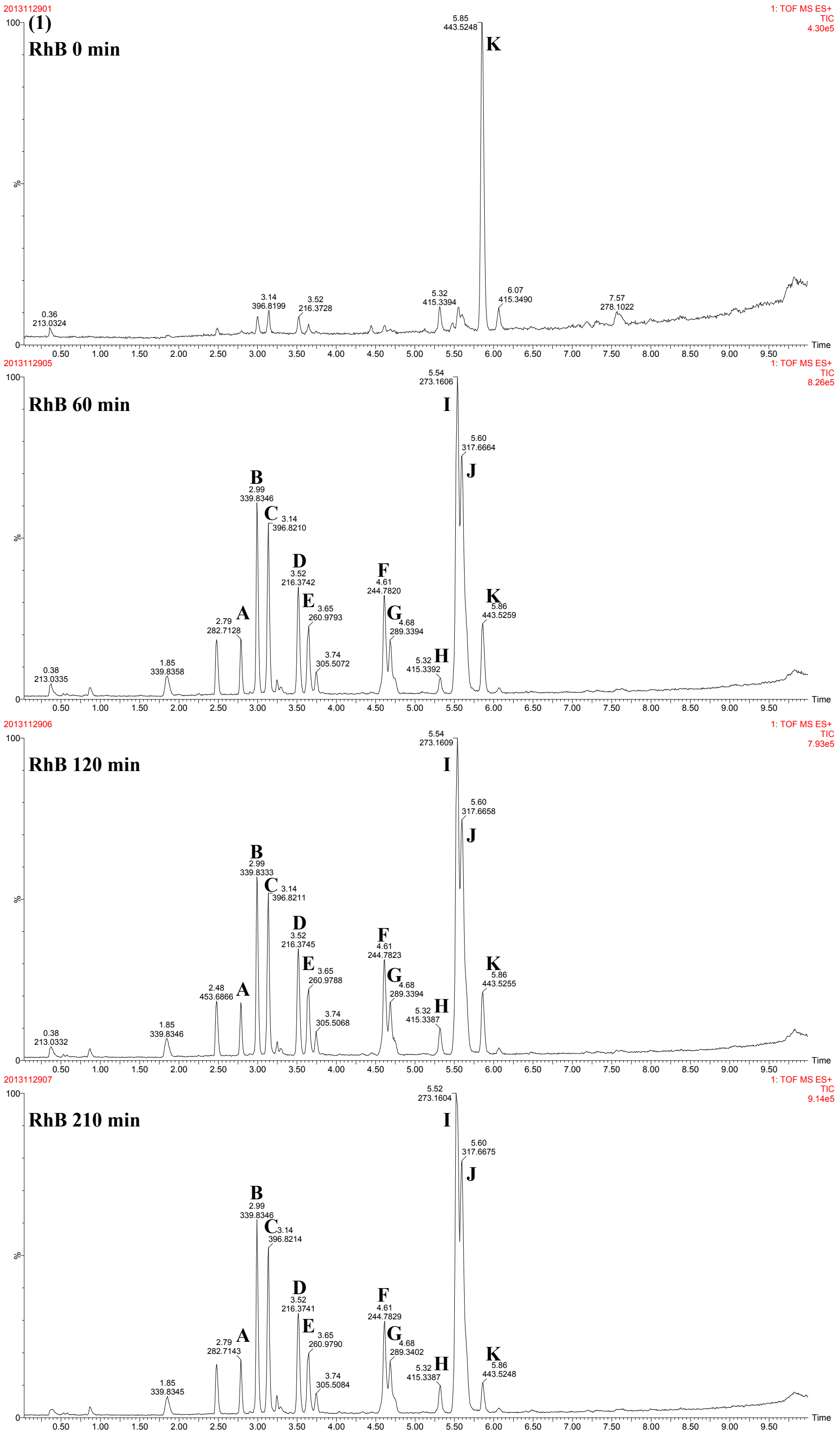

Fig. S6. Total ion chromatography of RhB and corresponding temporal photodegraded intermediate products in the presence of m-$KNbO_3$ NWs under UV.

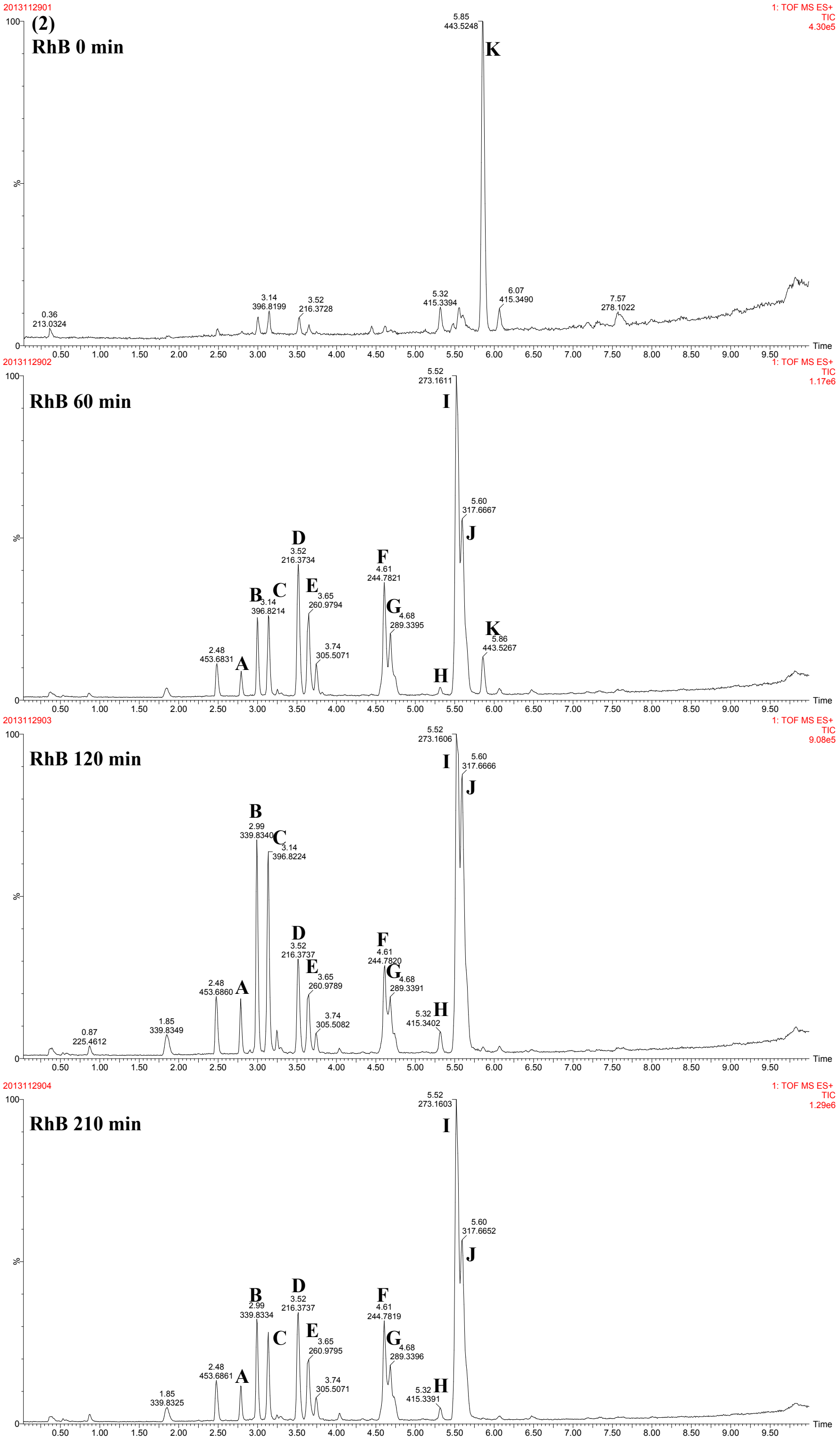

Fig. S7. Total ion chromatography of RhB and corresponding temporal photodegraded intermediate products in the presence of o-$KNbO_3$ NWs under UV.

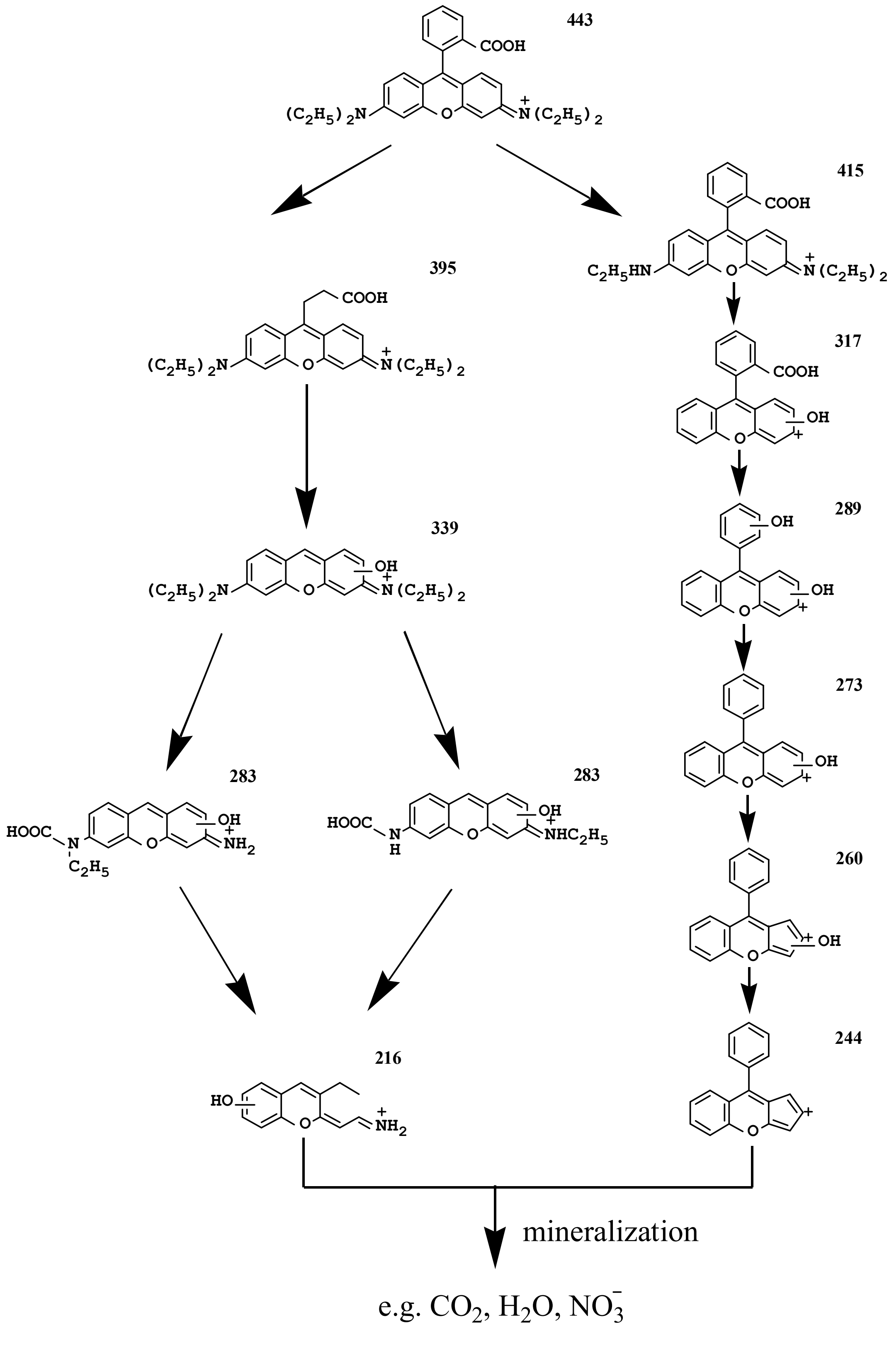

Fig. S8. Proposed photodegradation pathways of RhB by $KNbO_3$ NWs under UV.

References

[1] S. Kim, J. H. Lee, J. Lee, S. W. Kim, M. H. Kim, S. Park, H. Chung, Y. I. Kim, W. Kim, *J. Am. Chem. Soc.* **2013**, 135, 6.

[2] G. Z. Wang, S. M. Selbach, Y. D. Yu, X. T. Zhang, T. Grande, M. A. Einarsrud, *CrystEngComm* **2009**, 11, 1958.

[3] a) P. L. Galindo, S. Kret, A. M. Sanchez, J. Y. Laval, A. Yanez, J. Pizarro, E. Guerrero, T. Ben, S. I. Molina, *Ultramicroscopy* **2007**, 107, 1186; b) P. Galindo, J. Pizarro, S. Molina, K. Ishizuka, *Microsc. Anal.* **2009**, 23, 23.

[4] M. C. Payne, M. P. Teter, D. C. Allan, T. A. Arias, J. D. Joannopoulos, *Rev. Mod. Phys.* **1992**, 64, 1045.

[5] D. Hamann, M. Schlüter, C. Chiang, *Phys. Rev. Lett.* **1979**, 43, 1494.

[6] H. J. Monkhorst, J. D. Pack, *Phys. Rev. B* **1976**, 13, 5188.

[7] Z. Wu, R. Cohen, *Phys. Rev. B* **2006**, 73, 235116.

[8] L. Katz, H. D. Megaw, *Acta Cryst.* **1967**, 22, 639.

[9] K. Refson, P. R. Tulip, S. J. Clark, *Phys. Rev. B* **2006**, 73, 155114.

[10] F. Chen, J. C. Zhao, H. Hidaka, *Int. J. Photoenergy* **2003**, 05, 209.

[11] Z. He, C. Sun, S. G. Yang, Y. C. Ding, H. He, Z. L. Wang, *J. Hazard. Mater.* **2009**, 162, 1477.
